# Glioblastome révélé par un strabisme

**DOI:** 10.11604/pamj.2014.18.336.4552

**Published:** 2014-08-27

**Authors:** Zineb Jaja, Rajae Daoudi

**Affiliations:** 1 Hôpital des spécialités Rabat, Rabat, Maroc

**Keywords:** Glioblastome, strabisme, tumeur cérébrale, Glioblastoma, strabismus, brain tumor

## Image en medicine

Le glioblastome correspond à une tumeur cérébrale qui touche les astrocytes. C'est le cancer cérébral le plus fréquent (mais restant malgré cela rare) chez l'adulte, mais aussi le plus agressif. Le diagnostic repose sur l'interrogatoire qui cherche des signes neurologique et visuels évocateurs de la maladie. En cas de suspicion de pathologie cérébrale, un scanner et une Imagerie à Résonance Magnétique (IRM) cérébraux confirment le diagnostic et permettent de localiser l'emplacement de la tumeur. Une biopsie peut être effectuée après localisation de cette dernière si elle est accessible à un prélèvement. Cependant, le diagnostic est pratiquement sûr, dans la majeure partie des cas, avant d'effectuer la biopsie. Le traitement repose sur l'ablation de la tumeur par voie chirurgicale si celle-ci est accessible; la radiothérapie dans un second temps; la chimiothérapie. Nous representons le cas d'une patiente âgée de 50 ans ayant consulté pour une diplopie avec strabisme convergent dans un contexte d'altération de l’état general. Une IRM a été demandée; a objectivé un processus solido kystique au niveau du carrefour ventriculaire droit. Une biposie a été faite; l'examen histologique a révelé un glioblastome grade IV.

**Figure 1:**
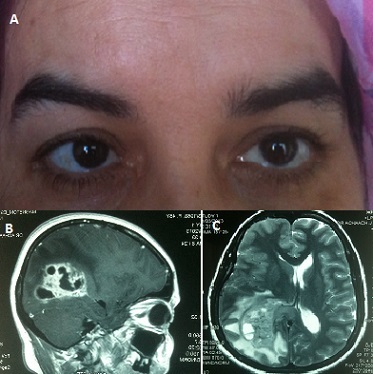
A) Photo montrant le strabisme convergent; B) coupe sagittale de l'IRM montrant le processus solido-kystique évocateur du glioblastome; C)IRM montrant le rehaussement après injection du gadolinium avec effet de masse au niveau ventriculaire et œdème périlesionel

